# 7-lncRNA Assessment Model for Monitoring and Prognosis of Breast Cancer Patients: Based on Cox Regression and Co-expression Analysis

**DOI:** 10.3389/fonc.2019.01348

**Published:** 2019-12-03

**Authors:** Huayao Li, Chundi Gao, Lijuan Liu, Jing Zhuang, Jing Yang, Cun Liu, Chao Zhou, Fubin Feng, Changgang Sun

**Affiliations:** ^1^College of Chinese Medicine, Shandong University of Traditional Chinese Medicine, Jinan, China; ^2^College of First Clinical Medicine, Shandong University of Traditional Chinese Medicine, Jinan, China; ^3^Department of Oncology, Weifang Traditional Chinese Hospital, Weifang, China; ^4^Department of Oncology, Affiliated Hospital of Weifang Medical University, Weifang, China; ^5^Chinese Medicine Innovation Institute, Shandong University of Traditional Chinese Medicine, Jinan, China

**Keywords:** breast cancer, univariate and multivariate Cox analyses, bioinformatic analysis, 7-lncRNA model, co-expression analysis

## Abstract

**Background:** Breast cancer is one of the deadliest malignant tumors worldwide. Due to its complex molecular and cellular heterogeneity, the efficacy of existing breast cancer risk prediction models is unsatisfactory. In this study, we developed a new lncRNA model to predict the prognosis of patients with BRCA.

**Methods:** BRCA-related differentially-expressed long non-coding RNA were screened from the Cancer Genome Atlas database. A novel lncRNA model was developed by univariate and multivariate analyses to predict the prognosis of patients with BRCA. The efficacy of the model was verified by TCGA-based breast cancer samples. Identified lncRNA-related mRNA based on the co-expression method.

**Results:** We constructed a 7-lncRNA breast cancer prediction model including LINC00377, LINC00536, LINC01224, LINC00668, LINC01234, LINC02037, and LINC01456. The breast cancer samples were divided into high-risk and low-risk groups based on the model, which verified the specificity and sensitivity of the model. The Area Under Curve (AUC) of the 3- and 5-year Receiver Operating Characteristic curve were 0.711 and 0.734, respectively, indicating that the model has good performance.

**Conclusion:** We constructed a 7-lncRNA model to predict the prognosis of patients with BRCA, and suggest that these lncRNAs may play a specific role in the carcinogenesis of BRCA.

## Introduction

Breast cancer (BRCA) is considered as the leading cause of death among gynecologic neoplasias. The treatment of BRCA has markedly improved due to advances in early screening and the development of anticancer strategies (1). However, breast cancer still exhibits a high recurrence rate (2). Studies have shown that the prognosis of breast cancer is affected by many factors like age, tumor size, grade, lymph node involvement, lymphovascular invasion, histology, hormone-receptor status, c-erbB2 status, and positive margins (3). Due to the pathogenic complexity of breast cancer, although many breast cancer prognostic biomarkers have been discovered, prognosis remains a difficult problem (4, 5). There is a need to construct a new breast cancer risk prediction model to improve the treatment of breast cancer patients. Due to the gene signature is yet limited in coding genes and microRNAs, to prove the necessity to develop the lncRNA model for predicting BRCA survival.

In the post-genomic era, many genome sequencing techniques have emerged (6). These tools provide new ideas and insights for tumor diagnosis and prognosis prediction. These next-generation sequencing methods and the data can thereby help better identify clinical biomarkers of cancer. The discovery of long non-coding RNA (lncRNA) has dramatically altered our understanding of cancer. The expression and dysregulation of lncRNAs is more cancer-type specific than the protein-coding genes (7). The latest research shows that lncRNAs play key roles in gene regulation and carcinogenesis, including proliferation, adhesion, migration, and apoptosis (8). Given the heterogeneity of BRCA and the complexity of non-coding RNAs, a panel of lncRNA biomarkers may be more precise and stable for BRCA prognosis (9). Shi et al. (10), based on The Cancer Genome Atlas (TCGA) database, constructed a 31-lncRNA model, which might be able to predict Overall Survival (OS) in patients with lung adenocarcinoma with high accuracy. Long et al. (11), by integrating the high-throughput data from the TCGA database, screened four genes (*CENPA, SPP1, MAGEB6*, and *HOXD9*) using univariate, Lasso, and multivariate Cox-regression analyses to develop the hepatocellular carcinoma prognostic model.

In this study, we screened breast cancer-associated differentially-expressed lncRNAs from the TCGA database and developed a new lncRNA model to predict the prognosis of patients with BRCA. It is well-known that lncRNAs could affect the function of proteins and cells directly or indirectly due to their involvement in the regulation of mRNA (12). Therefore, we have further explored the function of lncRNA in the model by studying the function of lncRNA-related mRNA. In summary, the use of lncRNA features provides a deeper insight into the prognosis of BRCA, which may be helpful in guiding the treatment.

## Materials and Methods

### Data Source

The lncRNA expression profiles and the corresponding clinical information from the patients with BRCA were obtained from The Cancer Genome Atlas (TCGA: https://cancergenome.nih.gov/) (13); a total of 1,208 samples, including 112 healthy and 1,096 BRCA samples. BRCA samples with incomplete prognostic information were excluded, and the average expression level was used as the final expression data of the same patient mRNA and lncRNA. A total of 1,076 BRCA samples were selected for further construction of the prognostic risk model and co-expression analysis. As the information was retrieved from the TCGA database, a public database, further ethical approvals do not apply to our research. Data collection and processing are in line with TCGA data policies for protecting human subjects (http://cancergenome.nih.gov/publications/publicationsguidelines).

### Identification of Differentially-Expressed lncRNAs and mRNAs

To identify the lncRNAs and mRNAs differentially expressed between the BRCA and the healthy samples, the downloaded lncRNA and mRNA data were standardized and differential-expression analysis was performed using the edgeR software package in the R software. The lncRNAs and mRNAs were differentially expressed with an absolute |logFC| > 2 and *p* < 0.01 were considered for subsequent analysis. The logFC indicates the fold change in the expression of each lncRNA and mRNA between BRCA and healthy breast tissue samples. Volcano plot of the differentially-expressed lncRNAs and mRNAs was obtained using the R software.

### Definition of the lncRNA-Related Prognostic Model

The lncRNA-related prognostic model was constructed based on the prognostic characteristics of lncRNA, and the correlation between overall survival (OS) and lncRNA expression levels was studied using univariate and multivariate Cox-regression analysis. Differences were assessed by univariate Cox proportional hazards regression analysis using R survival kits. For the association between expressed lncRNA and the overall survival, the lncRNA was considered significant when the *p*-value was <0.01 in the univariate Cox-regression analysis and was selected for multivariate Cox-regression analysis. Subsequently, multivariate Cox-regression analysis was performed to evaluate the contribution of genes as independent prognostic factors inpatient survival. A stepwise approach was used to further select the best model. A lncRNA-based prognostic risk score was calculated based on a linear combination of regression coefficients from the multivariate Cox-regression model (β) and its expression levels (10, 11).

$$\begin{array}{l} \text{Prognostic index}=\;\overset{N}{\underset{i=1}{\sum }}Exp_{i}\times \beta_{i} \end{array}$$

The Rpackage was used to find the optimal median threshold. According to the optimal median threshold, the survival data of 1,076 patients with BRCA were divided into low-risk and high-risk groups. Kaplan-Meier (KM) survival curves were generated to assess OS in low-risk or high-risk cases and time-dependent receiver operating characteristic (ROC) curve analysis was performed to calculate area under the curve (AUC) values to assess the predictive power of the model (14). Subsequently, we applied the model to patients with stage I, II, III, and Her2 positive BRCA to test the sensitivity and effectiveness of the model for survival prediction. In addition, we compared the predictive performance of 7-lncRNA model with traditional clinical risk factors (including age, TNM, stage, ER, PR, and HER2 status) by univariate and multivariate Cox analysis. First of all, univariate Cox analysis found factors closely related to the prognosis of patients. Then, the effects of many factors on survival time were analyzed at the same time, and the independent prognostic factors could be used to evaluate the survival of patients. *P* < 0.05 was used as the cutoff condition to verify the ability of the model to evaluate the prognosis and sensitivity of patients.

### Co-expression Method Predicts lncRNA-Related mRNAs

To better explore the function of the relevant lncRNAs in the risk assessment model, the related mRNAs were predicted by co-expression methods based on the Pearson correlation. The related mRNAs were screened for functional enrichment analysis according to |COR|> 0.25, *p* < 0.05. In addition, the lncRNA-mRNA co-expression network was visualized using Cytoscape.

### GO and KEGG Analysis of lncRNA-Related mRNA

To understand the underlying biological pathways between lncRNA and the related mRNAs, the database for annotation, visualization, and integrated discovery (DAVID) (http://david.abcc.ncifcrf.gov/) was used to perform functional enrichment analysis (15). Subsequently, lncRNA-related mRNAs were analyzed using the gene ontology (GO) database (http://www.geneontology.org). Finally, significantly enriched GO terms were selected to analyze their biological function. The Kyoto Encyclopedia of Genes and Genomes (KEGG; http://www.kegg.jp/) was used to perform the pathway enrichment analysis.

## Results

### Differentially Expressed lncRNAs and mRNAs in BRCA Patients

**Figure 1:**
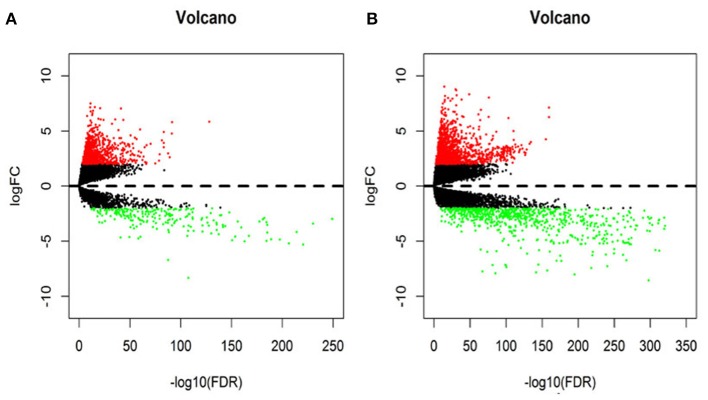
The volcano diagram about differentially expresses lncRNAs **(A)** and mRNAs **(B)** between breast cancer tissue and normal tissue samples. Red dots represent up-regulated RNA and green dots represent down-regulated RNA.

In this study, 1,208 samples were downloaded from the TCGA database and were used to identify differentially-expressed lncRNAs and mRNAs in BRCA patients, We analyzed the specific baseline clinical characteristic of 1,076 BRCA patients presented in Table 1. A total of 1,059 differentially expressed lncRNAs were obtained in accordance with |logFC|> 2 and *p* < 0.01.This included 842 upregulated lncRNAs and 217 downregulated lncRNAs (Figure 1A), and 2,138 differentially-expressed mRNAs included 1,375 upregulated mRNAs and 763 downregulated mRNAs (Figure 1B).

**Table 1:** Specific baseline clinical characteristic of 1,076 breast cancer patients.

	**1,076 breast cancer patients**
**Age**	
<60 years	572
≥60 years	504
**Stage**	
I	180
II	610
III	244
IV	19
Unknown	23
**Pathologic T stage**	
T1-2	897
T3-4	176
Unknown	3
**Pathologic N stage**	
N0-1	862
N2-3	194
Unknown	20
**Pathologic M stage**	
M0	896
M1	21
Unknown	159
**Estrogen receptor**	
Positive	790
Negative	237
Unknown	49
**Progesterone receptor**	
Positive	683
Negative	341
Unknown	52
**HER2**	
Positive	161
Negative	554
Unknown	361
**Survival time**	
≤ 1 years	185
1 years <	482
≤ 3 years	
3 years <	167
≤ 5 years	
>5 years	242

### Derivation of lncRNA Prognostic Model

**Figure 2:**
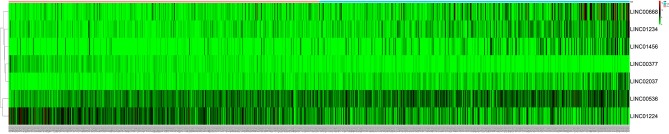
The heatmap of 7 independent breast cancer-related prognostic lncRNAs in the model. The color from green to red indicates a trend from low to high expression.

After excluding lncRNA without specific names and lack of corresponding studies, a total of 282 differentially-expressed lncRNAs remained for further study. Firstly, we performed a univariate Cox-regression analysis to study the correlation between differentially-expressed lncRNA and OS of BRCA patients. With a *p* < 0.01 as an identification standard, a total of 13 lncRNAs were obtained, which were significantly associated with OS in BRCA patients (Table 2). Subsequently, based on the primary screening using univariate Cox-regression analysis, we obtained seven lncRNAs that were used to construct a predictive model by performing stepwise multivariate Cox-regression analysis. They were LINC00377, LINC00536, LINC01224, LINC00668, LINC01234, LINC02037, and LINC01456 and the cluster dendrogram for these lncRNA is shown in Figure 2. The predictive model was characterized by the linear combination of the expression levels of the seven lncRNAs weighted by their relative coefficients from the multivariate Cox regression as follows:

**Table 2:** Thirteen prognosis-related lncRNAs obtained based on univariate Cox regression analysis (*P* < 0.01).

**Name**	**HR**	***z***	***p*-value**
LINC02037	1.243690056	4.120958497	3.77E−05
LINC01234	1.154170798	3.811036114	1.38E−04
LINC00668	1.105563899	3.700969179	2.15E−04
LINC01456	1.132314635	3.598894647	3.20E−04
LINC01592	1.238001411	3.465525511	5.29E−04
LINC02418	1.154200697	3.230285935	1.24E−03
LINC01854	1.221094553	2.881476012	3.96E−03
C6orf99	1.225252162	2.837096926	4.55E−03
LINC00536	1.117384364	2.763536856	5.72E−03
LINC01224	0.916544112	−2.70404596	6.85E−03
LINC02408	1.19605941	2.681361271	7.33E−03
LINC00377	0.748711948	−2.67297483	7.52E−03
LINC01574	1.145235673	2.589564334	9.61E−03

Prognostic index (PI) = (−0.2611 × expression level of LINC00377) + (0.0960 × expression level of LINC00536) + (−0.0966 × expression level of LINC01224) + (0.0738 × expression level of LINC00668) + (0.1014 × expression level of LINC01234) + (0.2020 × expression level of LINC02037) + (0.0627 × expression level of LINC01456).

Of these seven lncRNAs obtained by Cox-regression analysis, five (LINC00536, LINC00668, LINC01234, LINC02037, and LINC01456) showed positive coefficients, suggesting that these lncRNAs have a higher risk and their expression corresponds to the shorter OS in BRCA patients. In addition, the risk prediction correlation analysis between the seven lncRNAs is presented in Supplementary Figure 1. At the same time, the remaining two lncRNAs (LINC00377 and LINC01224) showed negative coefficients. Although the risk associated with these two lncRNAs is not higher, they are still important links in the prognosis model. These seven lncRNAs together constitute a prognostic model for patients with BRCA.

**Figure 3:**
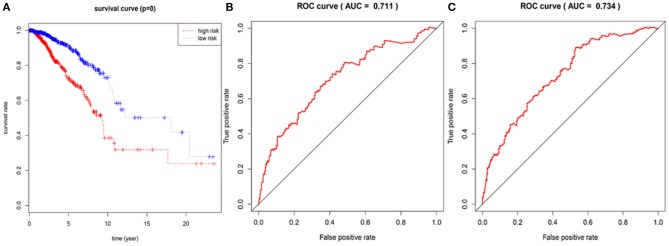
Assessment of prognostic risk in 1,076 breast cancer patients using an 7-lncRNA model, the Kaplan-Meier curve showed a poor prognosis in the high-risk group **(A)**. Time-dependent ROC curve analysis of 7-lncRNA model for survival prediction of breast cancer patients, ROC curve predicting 3 years survival rate (AUC = 0.711) **(B)**; ROC curve predicting 5 years survival rate (AUC) = 0.734) **(C)**.

In the 1,076 BRCA patients, the median of the prognostic score was obtained as the grouping threshold by calculating the risk scores for the expression of the seven lncRNAs. With a median PI as the group threshold, 538 patients with a prognostic score above the PI threshold were classified as high risk, while 538 patients below the PI threshold were assigned to the low-risk group. We found that Kaplan-Meier survival curve analysis of the high-risk and low-risk groups based on the prognostic risk model constructed by the seven lncRNAs showed that the overall survival rate of the high-risk group was lower, and the difference between the two groups was statistically significant (Figure 3A). Subsequently, the prognostic ability of the 7-lncRNA prognostic model was evaluated by calculating the AUC of the time-dependent ROC curve. Based on earlier results of the RUC curve, the higher the AUC, the better is the prediction performance of the model. For 3- and 5-year survival times, the AUC of the 7-lncRNA BRCA patient prognostic model was 0.711 and 0.734, respectively, indicating that the predictive model is highly sensitive and specific (Figures 3B,C).

**Figure 4:**
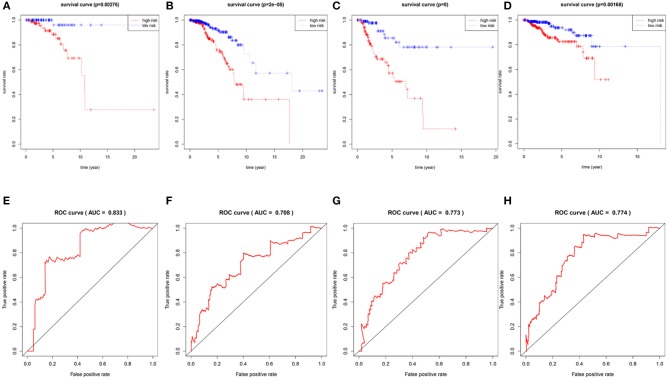
Verification the specificity and sensitivity of the 7-lncRNA prognostic model. The Kaplan-Meier curve of patients with stage I, stage II, stage III, and Her2-positive BRCA **(A–D)**; the ROC curve of the model at 3 years of OS with stage I, stage II, stage III, and Her2-positive BRCA, the AUC values were 0.883, 0.708, 0.773, 0.774 **(E–H)**.

To confirm the validity and sensitivity of the 7-lncRNA model for predicting survival, we applied the model to risk assessment in patients with stage I, stage II, stage III, and HER2 positive BRCA. Patients were divided into high-risk and low-risk groups using a median risk score (value = 0.965). The Kaplan-Meier curve results showed that the high-risk groups of patients with stage I, stage II, stage III, and Her2-positive BRCA were closely associated with poor prognosis (Figures 4A–D). In addition, the ROC curve indicated that the AUC values of the model were 0.883, 0.708, 0.773, 0.774 at 3 years of OS (Figures 4E–H), indicating that the 7-lncRNA model we constructed had certain specificity and sensitivity in evaluating the prognosis of patients with BRCA.

### Comprehensive Assessment of Model Predictive Performance and Routine Clinical Risk Factors

**Figure 5:**
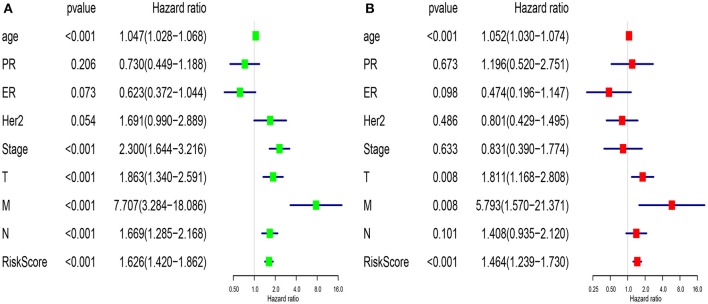
Univariate **(A)** and multivariate **(B)** analysis of clinic pathologic factors for overall survival of breast cancer patients from TCGA.

We compared the predictive performance of the 7-lncRNA model with conventional clinical risk factors, including age, TNM, Stage, ER, PR, and HER2 status. Univariate analysis found that age, Stage, TNM stage, and predictive performance of the 7-lncRNA model were closely related to prognosis (Figure 5A). Further multivariate analysis found that predictive performance of age, T, M, and 7-lncRNA models could be used as independent prognostic factors to assess patient outcomes (Figure 5B).

### Functional Assessment of lncRNA-Related mRNA

**Figure 6:**
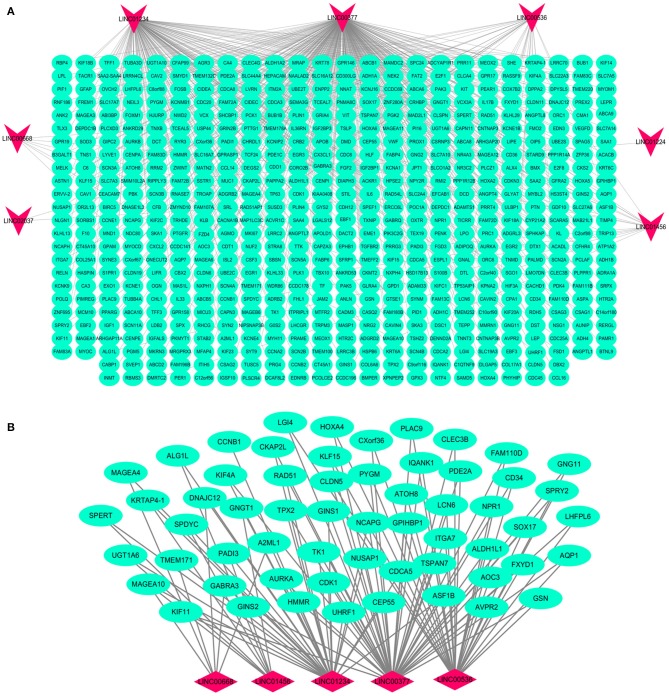
Interaction network map of lncRNAs in the model and related mRNAs. Visualization of the interaction of 7 lncRNAs and 592 mRNAs **(A)**; the mRNAs related to multiple lncRNAs expressions in the model **(B)**. Red nodes representing lncRNA and green nodes representing mRNA.

**Figure 7:**
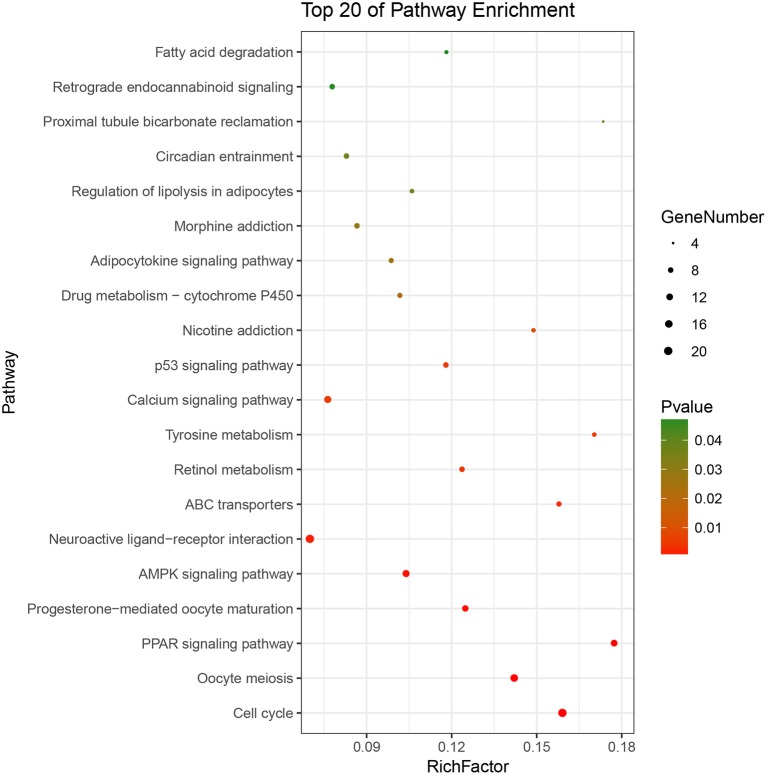
Pathways enrichment map of lncRNA-related mRNAs. Kegg terms were selected according to *P* < 0.05 and the most significant of the top 20 pathways were selected for visualization.

Based on the BRCA-related lncRNA and mRNA expression data from the TCGA database, co-expression analysis was performed using the Pearson correlation with |COR|> 0.25 and *p* < 0.05 as the cutoff. A total of 592 mRNAs were found to be closely related to the 7 lncRNAs (Figure 6). The functions of the lncRNA-related mRNAs were determined using DAVID bioinformatics resources 6.8. The results of GO analysis mainly include Biological Process (BP), Molecular Function (MF), and Cellular Component (CC) (Table 3). We selected the most significant 10 enrichment results in the 3 parts for analysis. The process of enrichment in BP mainly includes cell division, cell proliferation, cell adhesion, and DNA replication, processes that are closely related to the growth and proliferation of tumor cells. The characteristics of enrichment in MF are mainly ATP binding, calcium-ion binding, chromatin binding, and protein-kinase binding, and those related to CC are plasma membrane, cytosol, integral component of plasma membrane, and the extracellular region. Five hundred ninety-two mRNAs were mainly enriched in 20 signaling pathways (Figure 7), including cell cycle, oocyte meiosis, and other cell division and proliferation pathways; and cancer-related signaling pathways, such as PPAR signaling pathway, neuroactive ligand-receptor interaction, and p53 signaling pathway.

**Table 3:** Functional enrichment analysis of lncRNA-related mRNAs.

**Category**	**Term**	**Count**	***P*-Value**
Biological Processes	Cell division	45	1.13E−15
	Mitotic nuclear division	37	5.70E−15
	Positive regulation of cell proliferation	32	4.11E−05
	Cell proliferation	26	1.49E−04
	Cell adhesion	26	3.81E−03
	Response to drug	23	1.65E−04
	Sister chromatid cohesion	21	3.18E−11
	Cell surface receptor signaling pathway	19	1.90E−03
	DNA replication	17	2.07E−05
	G2/M transition of mitotic cell cycle	16	1.86E−05
Molecular Function	ATP binding	61	1.08E−02
	Calcium ion binding	35	5.20E−03
	Protein kinase binding	28	2.39E−05
	Chromatin binding	21	1.36E−02
	Microtubule binding	19	5.29E−05
	Transcriptional activator activity, RNA polymerase II core promoter proximal region sequence-specific binding	16	4.87E−03
	ATPase activity	15	1.19E−03
	Heparin binding	14	1.02E−03
	Transporter activity	14	7.65E−03
	Microtubule motor activity	12	2.41E−05
Cellular component	Plasma membrane	148	1.38E−02
	Cytosol	122	1.36E−02
	Extracellular region	79	2.15E−05
	Integral component of plasma membrane	67	2.85E−04
	Extracellular space	64	3.66E−04
	Centrosome	23	1.09E−02
	Microtubule	22	5.53E−04
	Apical plasma membrane	20	1.48E−03
	Midbody	17	1.83E−06
	Kinetochore	16	1.77E−08

*Enrichment analysis of biological processes, molecular function, and cellular component (P < 0.05)*.

**Figure 8:**
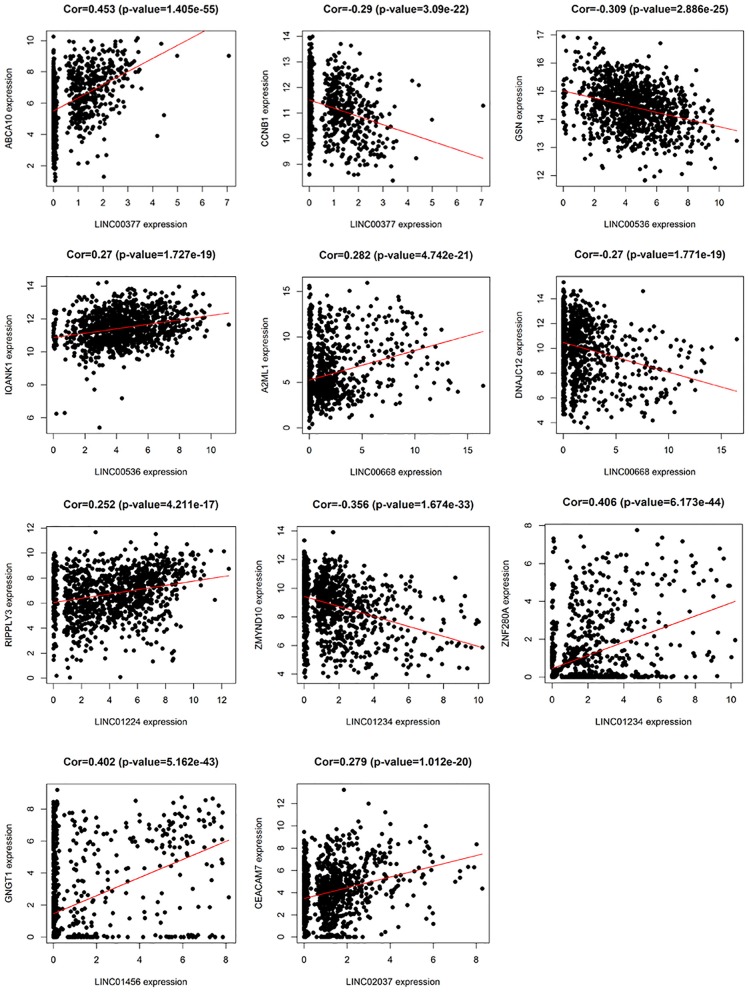
The relationship between 7 lncRNAs and related mRNAs (only the mRNAs that are most positively and negatively correlated with 7 lncRNAs are listed according to the correlation coefficient).

In addition, we identified up-regulated and down-regulated mRNA with the highest correlation coefficient with 7 lncRNAs, and obtained a total of 11 mRNAs, including *ABCA10, CCNB1, GSN, IQANK1, A2ML1, DNAJC12, RIPPLY3, ZMYND10, ZNF280A, GNGT1*, and *CEACAM7* (Figure 8).

## Discussion

BRCA is still one of the deadliest malignant tumors worldwide (16). Due to its complex molecular and cellular heterogeneity, the efficacy of existing breast cancer risk prediction models is unsatisfactory (17). High recurrence rate of breast cancer is one of the causes of high mortality. Therefore, in order to reduce mortality and improve the prognosis of BRCA, there is a need to construct a new breast cancer risk prediction model for clinical use. Clinicians should be able to develop individualized treatment plans for BRCA patients, establish strategies for prevention and early detection of BRCA recurrence, more frequently track high-risk populations, and perform regular clinical examinations for early diagnosis and recurrence of BRCA based on the predictions of the model.

In this study, BRCA-related differentially-expressed lncRNAs and mRNAs were obtained based on high-throughput RNA sequencing and clinical data of BRCA patients from the TCGA database. Subsequently, univariate and multivariate Cox analysis was performed to establish a risk model for predicting BRCA prognosis. Finally, BRCA prognostic risk prediction model was constructed using seven lncRNAs (LINC00377, LINC00536, LINC01224, LINC00668, LINC01234, LINC02037, and LINC01456). Applying the prognostic model to the TCGA BRCA dataset, breast cancer patients can be divided into high-risk and low-risk groups. The three- and 5-year AUC values for the time-dependent ROC curve were 0.771 and 0.734, respectively, indicating that the 7-lncRNA model has a good performance insurvival prediction. By exploring the correlation between differentially-expressed lncRNAs and mRNAs, lncRNA-related mRNAs were identified to further study the function of the 7 lncRNAs and the molecular mechanisms involved in breast cancer progression.

In the current study, among these 7 lncRNAs, LINC00668, LINC01234, and LINC01456 have been shown to play a role in the pathogenesis and prognosis of cancer. Zhao et al. (18) showed that in laryngeal squamous cell carcinoma, the expression levels of LINC00668 were associated with age, pathological differentiation degree, T stage, clinical stage, and cervical lymph node metastasis, and using a series of bioinformatics tools and *in vitro* experiments, proved that knockdown of LINC00668 can inhibit the proliferation, migration, and invasion ability of laryngeal squamous cell carcinoma cells. Zhang et al. (19) found that the expression of LINC00668 was negatively correlated with miR-297 expression in oral squamous cell carcinoma, and further found that LINC00668 promoted oral squamous cell carcinoma tumorigenesis via miR-297/VEGFA axis. In addition, Zhang et al. (20) found that knockdown of LINC00668 significantly inhibited the proliferation of gastric cancer cells *in vitro* and *in vivo*, and the significant increase in expression was associated with gastric cancer outcomes and prognosis. In our study, we found that the expression of LINC00668 is associated with *A2ML1* and *DNAJC12*; of which *A2ML1* has been shown to be closely related to the treatment of lung squamous cell carcinoma and can be used as a potential prognostic biomarker (21). Bubnov et al. (22) used genome-wide microarray Sentrix HumanWD-6V3 BeadChip (Illumina) to analyze gene expression pattern in 15 invasive adenocarcinoma samples and 15 healthy breast tissue samples, and found that *DNAJC12*, a member of the HSP40/DNAJ family, was significantly elevated. In addition, De Bessa et al. (23) found that *DNAJC12* is an estrogen target gene, its expression can be used as a marker of the ER activity, and that it may have a predictive value in response to hormonal therapy.

LINC01234 has been shown to be significantly associated with cancer treatment and prognosis in colon, gastric, and breast cancer (24–26). Chen et al. (27) found that LINC01234 expression was significantly upregulated in gastric cancer tissue and was associated with larger tumor size, advanced TNM stage, lymph node metastasis, and shorter survival. Furthermore, knockdown of LINC01234 induced apoptosis, arrested growth, and inhibited tumorigenesis in mouse xenografts. In our study, LINC01234 was found to be associated with *ZMYND10* and *ZNF280A*. *ZMYND10*, a candidate tumor suppressor gene, is frequently downregulated in nasopharyngeal carcinoma and many other tumors like gastric cancer, due to hypermethylation of the promoter (28). Functional evidence suggests that the *ZMYND10* gene inhibits tumor growth in animal experiments (29). According to reports, LINC01456 is a risk factor in ovarian cancer and is involved in the progression of ovarian cancer (30). In our study, we found a positive correlation between*GNGT1* and LINC01456 expression.

So far, no studies have reported any association between LINC00377, LINC00536, LINC01224, and LINC02037, and cancer. However, in our study, LINC00377 was found to be associated with expression of *ABCA10* and *CCNB1*. Ho et al. (31) found that *ABCA10* is involved in the pathogenesis of osteosarcoma, while Elsnerova et al. (32) found that the expression level of *ABCA10* was significantly associated with progression-free survival in ovarian cancer. *CCNB1* belongs to the highly conserved cyclin family and is significantly overexpressed in various cancer types. Ding et al. (33), showed that *CCNB1* had a significant predictive power in distant metastasis free survival, disease free survival, recurrence free survival, and overall survival of ER+ breast cancer patients. They also found that *CCNB1* was closely associated with hormone therapy resistance. LINC00536 was found to be associated with expression of *GSN* and *IQANK1*, a ubiquitous actin filament-cleaving protein and a well-known downregulated target in breast tumors (34). *GSN* overexpression studies in MDA-MB231 and MCF-7 cells indicated that increased expression of *GSN* can result in changes in cell proliferation and cell-cycle progression (35). In addition, Chang et al. (36) showed that LINC01224 is associated with the expression of *RIPPLY3*, LINC02037 is associated with the expression of *CEACAM7*, and *CEACAM7* is found to be a potential prognostic biomarker for colorectal cancer.

The use of the TCGA database broadens the range of models for cancer survival prediction. Compared with the previously constructed breast cancer lncRNA prognosis model (37, 38), the patient's sample data in the TCGA database is large, and the clinical information is complete, and there is complete prognosis survival data of breast cancer patients. The ROC curve can be used to assess the specificity and sensitivity of the model (AUC >0.7 indicates that the model has good sensitivity). The 7-lncRNA prognostic model we developed has the potential to predict the prognosis of patients with BRCA and is specific and sensitive. In addition, whether univariate or multivariate Cox-regression analysis, the predictive performance of the 7-lncRNA model we constructed can be a good assessment of prognosis, further indicating the evaluation value of the model. In addition, as the lncRNAs used in the model have a predictive effect on the prognosis of patients with BRCA, further experimental studies can be conducted to investigate the role of these lncRNAs in the pathogenesis of BRCA in order to provide new ideas and insights for treatment. However, current research still has some limitations, we attempted to validate the predictive performance of the 7-lncRNA model in other large breast cancer data sets. Unfortunately, due to the limitations of the clinical mutation information of breast cancer and patient prognosis information, we did not find a data set that met the verification requirements. So it is necessary to propose effective strategies such as including longer follow-up duration to validate the results and multiple regression modeling methods to improve the accuracy of the model.

## Conclusion

We constructed a 7-lncRNA prognostic model to reliably predict the prognosis of patients with BRCA, and these lncRNAs may play a role in the carcinogenesis of BRCA. Further functional studies are needed to elucidate the molecular mechanisms behind the roles of these lncRNAs in BRCA.

## Data Availability Statement

This manuscript contains previously unpublished data. The name of the repository and accession number are not available.

## Author Contributions

CS, HL, and CG conceived and designed the study. LL, JZ, and JY performed data analysis. CL, CZ, and FF contributed analysis tools. HL and CG wrote the paper.

### Conflict of Interest

The authors declare that the research was conducted in the absence of any commercial or financial relationships that could be construed as a potential conflict of interest.

## Supplementary Material

Figure S1 The risk prediction correlation analysis between the seven lncRNAs.

## Abbreviations

LncRNAs: long non-coding RNAs
BRCA: breast cancer
OS: overall survival
TCGA: the Cancer Genome Atlas
GO: gene oncology
KEGG: Kyoto Encyclopedia of Genes and Genomes
ROC: receiver operating characteristic
AUC: Area Under Curve
BP: biological process
CC: cellular component
MF: molecular function.
