# Supplementary figures and images for: 7-lncRNA Assessment Model for Monitoring and Prognosis of Breast Cancer Patients: Based on Cox Regression and Co-expression Analysis
Source: Front Oncol. 2019 Dec 3;9:1348. doi: 10.3389/fonc.2019.01348 (PMC6901675; doi:10.3389/fonc.2019.01348)

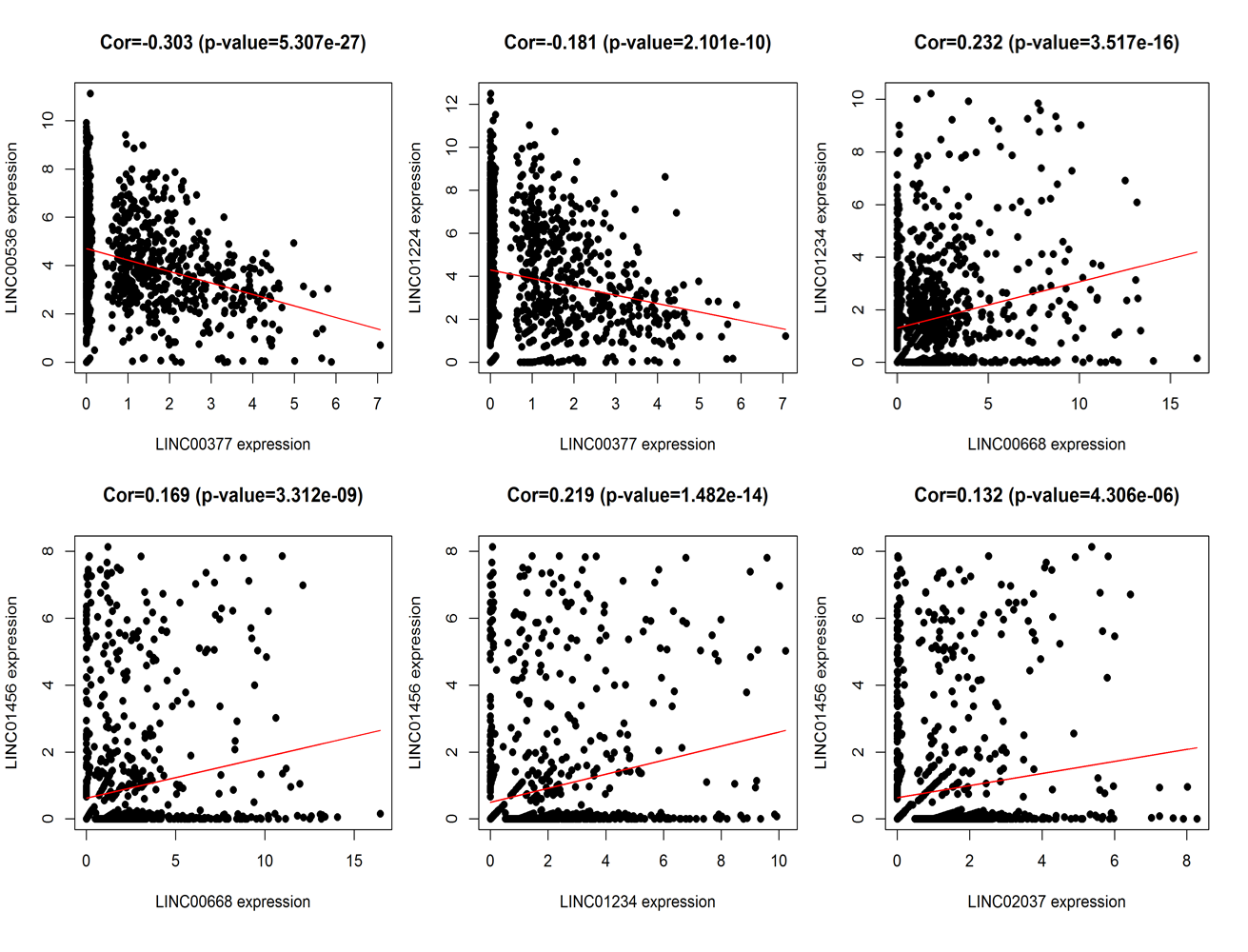

Supplement: Figure S1 — The risk prediction correlation analysis between the seven lncRNAs. [file Image_1.TIF]
